# Outpatient parenteral antibiotic therapy (OPAT) for the management of bronchiectasis

**DOI:** 10.1016/j.heliyon.2023.e19968

**Published:** 2023-09-09

**Authors:** Jacky Tu, Mohammed Al Harasi, Michael Pallin, Chris Daley, Benjamin A. Rogers, Paul T. King

**Affiliations:** a Monash Lung, Sleep, Allergy and Immunology, Monash Medical Centre (MMC), Clayton, Melbourne, Australia; b School of Clinical Sciences, Monash University, Monash Health, Clayton, Melbourne, Australia; c Monash Infectious Diseases, Monash Health, Clayton, Melbourne, Australia

**Keywords:** Bronchiectasis, Exacerbations, Outpatient, Parenteral, Antibiotics

## Abstract

**Background:**

Patients with bronchiectasis often require hospitalisation for the administration of intravenous antibiotics for the management of acute exacerbations. Increasingly, Outpatient Parenteral Antibiotic Therapy (OPAT) services have become available as a potential alternative for domiciliary management.

**Aims:**

This study assessed outcomes in both cystic fibrosis (CF) and non-CF bronchiectasis patients who received OPAT for the management of an acute exacerbation of bronchiectasis.

**Methods:**

A retrospective study of consecutive subjects was done in both CF and non-CF groups in a large metropolitan Health Service in Australia from 2016 to 2022.

**Results:**

There were 51 episodes of care in the non-CF group (22 subjects) and 73 episodes in the CF group (13 subjects). The non-CF group were nearly all treated with once daily domiciliary intravenous (IV) ceftriaxone (49/51 episodes) for a duration of 9.1 ± 3.0 days (mean and standard deviation (SD)) via a peripherally inserted venous canula (84% of episodes). In contrast, the CF group generally received dual IV antibiotics (64% of episodes), with an average duration of 16.8 ± 6.3 days via central venous access (100%). In the non-CF group, the admission rate to hospital after 1 month was 9.6% and in the CF group was 0%. At 3 and 6 months the readmission rate for the non-CF group was 15.7% and 19.6% and CF group was 21.9% and 31.5%. There was a low rate of complications for the OPAT admissions (2% for the non-CF group and 7% for CF group).

**Conclusions:**

OPAT is a viable alternative for the management of bronchiectasis exacerbations.

## 1 Introduction

As the utilisation of hospital inpatient services has rapidly escalated, there has been an urgent need to establish new models of patient care. Outpatient Parenteral Antibiotic Therapy (OPAT) has emerged as a viable alternative for selected patients. OPAT services can lead to cost-savings over inpatient care [1] and are often preferred by patients [2]. The COVID-19 pandemic has created significant impetus for the outpatient management of previously hospitalised patients to free up healthcare service capacity, minimise the complications of bed-based care and protect vulnerable patients from nosocomial exposure to COVID-19 [3].

In Australia, the OPAT model of care has most commonly been used in two groups: a) admitted inpatients to facilitate earlier discharge when patients are clinically stable but need long-term treatment that has traditionally been administered in hospital (e.g., intravenous (IV) antibiotics for complicated infections) and b) to support discharge for patients presenting the Emergency Department(ED) with uncomplicated infections such as lower-limb cellulitis. Subjects are typically carefully selected and assessed by the clinical team. In more recent times, this model of care has been used to completely replace bed-based care with direct admissions from the community for IV antibiotic administration for suitable community dwelling patients.

Subjects with a primary diagnosis of suppurative lung disease may be suitable for OPAT although their functional status varies more than some other groups, making clinical assessment of their underlying condition important. Patients with cystic fibrosis (CF) often require hospital admission for inpatient management with IV antibiotics for acute infective exacerbations for CF-related bronchiectasis [4]. As these patients are young, they are generally strongly motivated to be able to keep working/attending educational facilities. A standard admission may comprise 1–2 weeks in hospital with IV antibiotics and input from a multi-disciplinary team (e.g., physiotherapy, dietician, and relevant specialist services) then OPAT with administration of IV antibiotics and home physiotherapy. More recently, patients have been directly admitted to the OPAT service without a preceding inpatient admission. In this model, subjects are assessed at a hospital clinic and then commenced on IV antibiotics through direct admission to OPAT for treatment of their CF-related bronchiectasis exacerbations. In this scenario, the patient has home visitations from nursing staff to administer the antibiotics and remote physiotherapy and this model has proved to be reasonably successful [[5], [6], [7], [8]].

Non-CF bronchiectasis is a prevalent respiratory condition often requiring treatment with IV antibiotics and is also potentially suitable for management by OPAT [9]. The literature describing home parenteral antibiotic usage in non-CF bronchiectasis is limited [[10], [11], [12]]. A comprehensive study by Bedi et al. described domiciliary antibiotic use in a cohort of subjects with a proportion initially treated as inpatients [11]. A variety of different antibiotics were used and the readmission rate at one month was 14%.

In our health service, we have developed a pathway for the treatment of bronchiectasis exacerbations (defined as an increase in their daily respiratory symptoms) where subjects are managed using OPAT. This was initially established with patients with CF-related bronchiectasis before being expanded to include the management of non-CF bronchiectasis subjects.

The aim of our study was to evaluate the role of OPAT for the management of exacerbations of non-CF bronchiectasis and how this compares to the management of CF-related bronchiectasis cohort. The primary outcome evaluated was hospital readmissions rate for a respiratory-related illness at 1, 3 and 6 months, including admissions to the Intensive Care Unit (ICU), following their OPAT admission. A secondary outcome of the study was to evaluate tolerability of the prescribed IV antibiotic therapy.

## 2 Materials and methods

A retrospective study of patients with CF and non-CF bronchiectasis was performed at Monash Health in Australia between January 2016 and April 2022. Monash Health serves a population of >1.5 million people and is comprises four acute adult hospitals. The health service has a large OPAT program with ambulatory clinics at all four sites and home-visiting teams [13]. The Monash CF service is one of the two dedicated state-wide services in Victoria, with a broader catchment area that consists of the majority of eastern metropolitan and regional Victoria. All potentially eligible subjects were identified from a consecutive list provided by the treating respiratory physicians and the Respiratory Medicine database. Inclusion criteria included all patients with CF or non-CF related bronchiectasis who had received OPAT for the management of an exacerbation (defined as an increase in daily respiratory symptoms) [14]. All subjects who received IV antibiotics as a hospitalised inpatient prior to admission to the OPAT program were excluded. This research project was approved by the Monash Health Ethics Committee (number 66238). This study was approved by the Ethics committee as a quality control audit and therefore formal individual patient consent was not required.

These patients were assessed by their treating Respiratory Physician (MP, CD or PK) as having significant respiratory symptoms attributed to an exacerbation of bronchiectasis and were appropriate for a trial of IV antibiotics and clinically stable to be managed in an ambulatory environment (rather than requiring inpatient admission). All the non-CF subjects had received oral antibiotics in the month prior to initiation of OPAT, and none had resolution of their exacerbation. Subjects had a sputum sample taken at time of admission for microbial analysis and were referred to the OPAT service at Monash Health.

All patients referred to this program undergo a nursing and medical assessment by the OPAT staff. In addition to assessment of the patient's clinical status and general suitability for OPAT care, the patient's home environment is confirmed to be concordant with program requirements. Subjects had IV access facilitated (i.e., insertion of peripheral vein canula, peripheral inserted central catheter (PICC) or used an established long-term IV port). The first dose of antimicrobial is administered in an ambulatory clinic. Subsequent doses are administered either within the clinic or a suitable community location (e.g., patient's home, workplace, education location). The decision on location is based on patient preference, suitability of the patient and environment for community-based administration and nursing capacity. Therapy can be administered up to two times a day. Based on local procedure, a portion of patients with CF antibiotic therapy is administered by the patient or carer, rather than nursing staff. For the CF cohort, the duration of antibiotic treatment was determined by improvement in sputum volume, exercise tolerance and pulmonary function testing with IV antibiotic therapy by the treating CF multidisciplinary (medical, nursing, allied health) team. For the non-CF cohort, the clinical decision on the therapy duration was based on duration of previous courses of therapy, patient and nursing staff reported symptom improvement on therapy and a clinical assessment (routinely undertaken on Day 7 of treatment). Patients were administered short course systemic prednisolone if they had wheeze at the time of their exacerbation or there was a diagnosis of allergic bronchopulmonary aspergillosis. Most CF patients also had weekly chest physiotherapy visits. Subjects were also routinely assessed by hospital medical staff on Day 7 and at the end of treatment.

The clinical correspondence, Hospital Medical Record, Respiratory Function Laboratory Data and Monash Pathology records were assessed for relevant details about admissions, demographic and health data, laboratory results and readmission rates/complications. The distance between a patient's place of residence and the primary hospital ambulatory OPAT Clinic was also measured.

The demographic, clinical and outcome variables of interest were summarised using descriptive statistics. Continuous variables were expressed as means and standard deviation (SD) and categorical variables as number (percentage) as appropriate. The CF and non-CF groups were compared using unpaired *t*-test or contingency analysis (Fisher's exact test) as appropriate. Data was analyzed using Excel and Prism software as appropriate.

## 3 Results

During the study period, 229 OPAT potentially eligible episodes of care were identified; 124 presentations met inclusion criteria and were analyzed (Table 1). There were 22 subjects with non-CF bronchiectasis with 51 OPAT admissions and 13 CF subjects with 73 OPAT admissions. The average age of the non-CF cohort was older (66.0 ± 12.8 years) compared with the non-CF cohort (34.3 ± 11.8 years) (p < 0.001). The non–CF–cohort had better lung function testing and less extensive disease on computed (CT) scanning (Table 1) (all parameters p < 0.001). Patients treated with CF lived a significant distance from their hospital's ambulatory OPAT Clinic compared with those treated for a non-CF related bronchiectasis exacerbation (p < 0.001). This difference was explained by the Monash CF service covering most of eastern Victoria and the CF-related OPAT services being managed through a single ambulatory site (Monash Medical Centre). In both groups, most patients had more than one other medical comorbidity (Supplementary Table 1).

**Table 1:** Patient details.

Patient DetailS	Non-CF group (n)	CF group (n)
Episodes of Care	51	73
Individual Patients	22	13
Age (years)	66.0 ± 12.8	34.3 ± 11.8
Sex (Male)	7/22 (31.8%)	4/13 (30.8%)
Sex (Female)	15/22 (68.2%)	9/13 (69.2%)
Distance (km) from OPAT Admission Site	11.0 ± 5.7	48.4 ± 64.8
≥1 comorbidity	20 (90.9%)	11 (84.6%)
Mean FEV1 (%)	72.7 ± 23.4	57.5 ± 15.1
Mean FVC (%)	87.8 ± 14.1	75.7 ± 13.3
Mean FER	0.65 ± 0.12	0.73 ± 0.13
% FER < 0.7	12 (63.2%)	6 (46.2%)
Mean no. of lobes with bronchiectasis on CT	2.5 ± 1.1	3.5 ± 1.0

Abbreviations: CF, Cystic fibrosis; CT, computed tomography scan; FEV1, forced expiratory volume in 1 s; FER, forced expiratory ratio; FVC, forced vital capacity; n, number; OPAT, outpatient parenteral antibiotic therapy; SD, Standard Deviation.

Results expressed as mean ± SD or number (%).

The sputum microbiology was quite different between the two groups. In the non-CF group, normal upper respiratory tract flora was reported in 41/51 (80.4%) episodes of care. The most common isolated pathogen was *Haemophilus influenzae* followed by *Aspergillus fumigatus*. In contrast, in the CF subjects the most common pathogens were *Pseudomonas aeruginosa*, then *A*. *fumigatus, Stenotrophomonas maltophilia* and *Staphylococcus aureus* (Supplementary Table 2).

Subjects with non-CF related bronchiectasis had a shorter length of stay (9.1 ± 3.0 days versus 16.8 ± 6.3 days, p < 0.001) and less antibiotic days than the CF cohort (Table 2). The non-CF group had all 51 episodes of care treated with a single antibiotic regimen whilst only 23/73 episodes in the CF group had single antibiotic usage (p < 0.001). Treatment regimens were different between the two groups (Table 2). The great majority of the non-CF group had their antibiotics administered using a peripheral venous cannula (84%); subjects who required more than 14 days of IV therapy or who had poor venous access had a PICC through the Radiology service. None of the CF subjects had a peripheral venous cannula; most had a long-term port for access and in the rest a PICC line was used. None of the non-CF group received domiciliary physiotherapy treatment, whilst most of the CF group received remote weekly physiotherapy reviews (Table 2).

**Table 2:** Treatment.

Treatment^†^	Non-CF group (n)	CF group (n)
Duration of IV therapy (days)	9.1 ± 3.0	16.8 ± 6.3
Completed prescribed course of IV antibiotic therapy	51 (100%)	73 (100%)
Antibiotic Days (Total Days)	466	2024
Missed OPAT Clinic Reviews/Doses	2 (3.9%)	3 (4.1%)
Outpatient Physiotherapy reviews	0 (0%)	50 (68.5%)
**Single Antibiotic Regimen**	51/51 (100%)	25/73 (34.2%)
Benzylpenicillin	2	0
Ceftazidime	0	11
Ceftriaxone	49 (96.1%)	0
Flucloxacillin	0	3
Meropenum	0	1
Piperacillin-Tazobactam	0	9
Tobramycin (IV)	0	1
Vancomycin	0	1
**Dual Antibiotic Regimen**	0/51 (0%)	48/73 (65.8%)
Cefepime + Tobramycin	0	2
Ceftazidime + Tobramycin	0	23
Cephazolin + Flucloxacillin	0	1
Meropenum + Ceftazidime	0	4
Meropenum + Tobramycin	0	4
Piperacillin-Tazobactam + Ciprofloxacin	0	2
Piperacillin-Tazobactam + Tobramycin	0	11
**Method of IV Administration**^**†**^		
Peripheral IV Cannula	43 (84.3%)	0
PICC	8 (15.7%)	26 (35.6%)
Port	0	47 (64.4%)

Abbreviations: ADR, Adverse Drug Reaction; IV, intravenous; PICC, peripherally inserted central catheter.

All subjects in the non-CF group were treated with one antibiotic and in 49/51 admissions this was ceftriaxone (two episodes were treated with benzylpenicillin), (Table 2). Most CF subjects received combination IV antibiotics. Ceftazidime or piperacillin-tazobactam in combination with tobramycin were the most commonly prescribed.

Subjects were admitted to the OPAT service as an alternative to inpatient hospitalisation. We assessed the rate of subsequent hospital admission at 1, 3 and 6 months after the completion of OPAT treatment (Table 3). In the non-CF group, 9.8% of subjects required inpatient admission in the first month; these subjects were referred by the OPAT Program due to concerns that patients had failed domiciliary therapy and required hospitalisation for further management, including access to inpatient physiotherapy, nursing, and medical services. None of the CF subjects required readmission in the first month (p = 0.010). In contrast, the rate of hospitalisation within 3 (21.9%) and 6 months (31.5%) was higher amongst the CF-related bronchiectasis cohort compared to the non-CF related bronchiectasis (15.7% and 19.6% respectively (p = 0.26 at 3 months and p = 0.10 at 6 months). In both cohorts, most patients were treated with IV antibiotic therapy. A mean hospital length of stay of six days was reported in both cohorts. None of these readmissions over the subsequent 6 months required treatment in the intensive care unit; nor were there any major adverse outcomes reported in both the non-CF and CF group.

**Table 3:** Outcomes.

OUTCOMES	Non-CF group (n)	CF group (n)
Hospitalisation within 1 month	5 (9.8%)	0 (0%)
Hospitalisation within 3 months	8 (15.7%)	16 (21.9%)
Hospitalisation within 6 months	10 (19.6%)	23 (31.5%)
Received IV antibiotics	9/10 (90%)	20/23 (87.0%)
Transferred to OPAT post-IP admission	3/10 (30%)	10/23 (43.5%)
Length of inpatient stay (days)	6.6 ± 3.3	6.7 ± 3.4
Length of inpatient + OPAT admission (days)	7.6 ± 6.3	11.6 ± 7.8
Admitted to ICU	0 (0%)	0 (0%)
Any ADR	1/51 (2.0%)^a^	5/73 (6.8%)^b^

Abbreviations: ADR, adverse drug reaction; ICU, Intensive Care Unit; IP, inpatient; IV, intravenous.

a One episode of dizziness.

b Two episodes of diarrhea, one episode of nausea/vomiting, one episode of headache and one episode of line-related thrombosis.

OPAT was generally very well tolerated in both groups with a low incidence of adverse effects or complications (Table 3). In the non-CF group, there was one report of dizziness and two missed doses/visits; all episodes of care had completion of the prescribed course of IV antibiotics. In the CF group there were five reports of adverse drug reaction (7%) including gastro-intestinal upset, headache, and one occurrence of line-related thrombosis; there were three missed doses/visits and all episodes of care had completion of the prescribed course of antibiotics.

## 4 Discussion

This study demonstrates that OPAT with exclusive domiciliary treatment can be used to successfully manage non-CF bronchiectasis patients with minimal complications and a low readmission rate. Nearly all patients were treated with once daily Ceftriaxone mostly given by a peripheral venous cannula. In contrast, subjects with CF-related bronchiectasis had longer admissions, with administration of multiple antibiotics given via central access.

Home IV antibiotics have been well-established for the management of cystic fibrosis, particularly as follow up therapy in patients who have been hospitalised. In contrast there is a lack of published literature describing the use of OPAT in non-CF bronchiectasis. Bedi et al. described 84 episodes of domiciliary care in the UK [11]. This cohort was treated with ten different antibiotic regimes of which the most common was Ceftazidime with improvement in clinical and laboratory parameters from day 1 to day 14. The readmission rate at 30 days was 14%. Antibiotics were administered by a PICC line. There was no information provided regarding whether patients were administered antibiotics once a day or multiple times a day. A Spanish study assessed the role of OPAT in the management of bronchiectasis; 22 episodes of care with exclusive domiciliary treatment were listed. Most subjects were treated with a variety of dual antibiotics and subjects all appeared to have multiple treatments daily. The authors reported that this approach was safe and reduced hospitalisation [10]. A paediatric study assessed the use of OPAT in children who were all initially treated as in patients. Subjects were treated for a mean duration of 14 days with a variety of antibiotics (specific numbers were not listed); outcomes were similar to patients only treated in hospital [12].

In our non-CF bronchiectasis cohort nearly all episodes of care were treated with once daily IV Ceftriaxone. This required running an IV infusion over the space of 40–60 min with no need for therapeutic drug monitoring. Subjects generally had a very low incidence of resistant bacteria on sputum analysis and generally only had mildly reduced lung function and were all living independently consistent with good functional status. In contrast in our CF group, subjects had lower lung function with a much higher prevalence of antimicrobial-resistant bacteria; most of the CF group were treated with at least two parenteral antibiotics. Patients in the non-CF group had a shorter length of stay (LOS) compared to the CF subjects and that of the Bedi et al. group (14 days) [11]; the optimal LOS is not clearly defined for these subjects but important factors to consider are duration of treatment to reduce bacterial infection, patient convenience and overuse of antibiotics. The patients in the non-CF group did have a higher rate of readmission within one month, this could be related to the shorter duration of antibiotic therapy.

The IV access type used for parenteral therapy has implications for the clinical utility of parenteral therapy. In our non-CF bronchiectasis cohort, most episodes of care were administered using a peripheral venous cannula, inserted by medical or nursing staff. This significantly simplifies patient admission to the OPAT program and may reduce the cost of care. Within our healthcare service, insertion of a PICC is undertaken by the Interventional Radiology service. Delays in accessing this service are a barrier to rapid admission to the OPAT program. Additionally, PICC confer a higher risk of complications include line-related thrombosis and infection compared with peripheral venous cannulas [15]. None of the CF cohort had their antibiotics given via a peripheral venous catheter; instead, a longstanding port (for most) or a PICC line was used.

The CF direct OPAT model of care has been established as a part of our local healthcare service's CF program, modelled after successful international ambulatory OPAT programs for CF-related bronchiectasis exacerbations [16]. Physiotherapy is a standard part of clinical care, which is generally provided weekly as part of our CF Service during their treatment. In contrast, there is no provision of physiotherapy services for non-CF bronchiectasis outpatients in the studied health service. Patients who have marked sputum production and poor clearance, particularly those with more advanced disease may not be suitable for management with OPAT.

The cost of OPAT for the treatment of bronchiectasis-related exacerbations has been estimated to be 0.40–0.56 of the total cost of an equivalent inpatient hospital admission in other jurisdictions [17]. In addition with COVID, there have been significant issues with bed access with significant emphasis being placed on the desirability of avoiding direct hospitalisation. Direct OPAT admissions, therefore, not only provide an opportunity to deliver cost-effective care to patients but creates greater capacity for healthcare services to have provide care to other patients requiring hospital inpatient admission.

The use of IV antibiotics may potentially have some benefits for improved antimicrobial stewardship. Many of the patients in the non-CF had been treated with prolonged courses of oral antibiotics (e.g., 5–6 courses over several months) before receiving OPAT therapy. The use of a single course of OPAT may potentially have a benefit of decreased antibiotic usage.

The main limitation of this study is that it was retrospective. We did not directly assess whether patients had immediate clinical benefit with the use of antibiotics but the main outcome that we were interested in assessing whether this was a viable alternative to inpatient admission in the non-CF group and there was a low rate of readmission, particularly in the first month. The data from the CF group did not provide any major new insights by itself but served as a valuable comparison group for the non-CF subjects.

## 5 Conclusion

This study demonstrates that OPAT is a viable alternative to inpatient admission in subjects with non-CF bronchiectasis exacerbations. The regime used for the most patients was once daily IV Ceftriaxone given by a peripheral venous catheter. There were minimal side effects and a low rate of readmission. In selected patients OPAT is a viable treatment option.

## Author contribution statement

Paul King: Conceived and designed the experiments; Analyzed and interpreted the data; Contributed reagents, materials, analysis tools or data; Wrote the paper. Jacky Tu; Mohammed Al Harasi: Performed the experiments; Analyzed and interpreted the data. Michael Pallin; Chris Daley: Contributed reagents, materials, analysis tools or data. Ben Rogers: Conceived and designed the experiments; Analyzed and interpreted the data.

## Funding statement

A/Prof. Rogers is supported by a NHMRC Emerging Leadership Grant.

## Data availability statement

Data included in article/supplementary material/referenced in article.

## Declaration of competing interest

The authors declare that they have no known competing financial interests or personal relationships that could have appeared to influence the work reported in this paper.
