# Supplementary material for: Outpatient parenteral antibiotic therapy (OPAT) for the management of bronchiectasis
Source: Heliyon. 2023 Sep 9;9(9):e19968. doi: 10.1016/j.heliyon.2023.e19968 (PMC10559547; doi:10.1016/j.heliyon.2023.e19968)
Supplement: Multimedia component 1 [file mmc1.docx]

**Supplementary Data**

**Supplementary table 1: Comorbidities**

| Comorbidities | Non-CF group (n=22) | CF group (n=13) |
| --- | --- | --- |
| No comorbidities | 2 | 2 |
| >1 comorbidity | 20 | 11 |
| Diabetes Mellitus | 1 | 1 |
| Hypertension | 3 | 0 |
| Chronic kidney disease | 1 | 0 |
| Cardiovascular disease | 3 | 0 |
| Anxiety/depression | 1 | 0 |
| Asthma | 3 | 11 |
| Inflammatory bowel disease | 1 | 0 |
| Chronic obstructive pulmonary disease | 5 | 0 |
| Rheumatoid arthritis | 1 | 0 |
| Chronic sinusitis | 1 | 0 |

**Supplementary Table 2: Sputum microbiology**

| Sputum MCS – Pathogens (1^st^ OPAT Admission) | Non-CF group (n=22) | CF group (n=13) |
| --- | --- | --- |
| No growth | 2 | 0 |
| Normal upper respiratory flora | 15 | 0 |
| Bacterial |  |  |
| *Haemophilus influenzae* | 5 | 1 |
| *Klebsiella pneumoniae* | 0 | 1 |
| *Moraxella* | 1 | 0 |
| *Pseudomonas aeruginosa* | 2 | 9 |
| *Serratia marcescens* | 1 | 0 |
| *Staphylococcus aureus* | 1 | 4 |
| *Stenotrophomonas maltophilia* | 0 | 4 |
| *Streptococcus pneumoniae* | 1 | 0 |
| Fungal |  |  |
| *Aspergillus fumigatus* | 3 | 6 |
| *Expophiala species* | 0 | 1 |
| Yeast species | 4 | 9 |
| Mycobacterial |  |  |
| *Mycobacterium abscessus* | 1 | 0 |

| Sputum MCS – Pathogens (Across all OPAT Admissions) | Non-CF group (n=51) | CF group (n=73) |
| --- | --- | --- |
| No growth | 2 | 0 |
| Normal upper respiratory flora | 41 | 0 |
| Bacterial |  |  |
| *Bulkholderia cepacia complex* | 0 | 6 |
| *Enterobacter clocae complex* | 0 | 2 |
| *Haemophilus influenzae* | 19 | 3 |
| *Klebsiella pneumoniae* | 0 | 6 |
| *Moraxella* | 3 | 0 |
| *Pseudomonas aeruginosa* | 2 | 63 |
| *Serratia marcescens* | 1 | 0 |
| *Staphylococcus aureus* | 1 | 16 |
| *Staphylococcus aureus* – methicillin-resistant (MRSA) | 0 | 1 |
| *Stentotrophomonas maltophilia* | 0 | 22 |
| *Streptococcus pneumoniae* | 2 | 0 |
| Fungal |  |  |
| *Aspergillus fumigatus* | 17* | 38 |
| *Expophiala species* | 0 | 2 |
| Yeast species | 7 | 35 |
| Mycobacterial |  |  |
| *Mycobacterium abscessus* | 1 | 0 |

*Only one patient with documented ABPA
